# A quantitative method to compare regional tumor contrast between prone and supine breast MRI

**DOI:** 10.3389/fonc.2026.1479189

**Published:** 2026-04-27

**Authors:** Brook K. Byrd, Venkataramanan Krishnaswamy, Misty J. Fox, Jiang Gui, Roberta DiFlorio-Alexander, Keith D. Paulsen, Richard J. Barth, Timothy B. Rooney

**Affiliations:** 1 Thayer School of Engineering, Dartmouth College, Hanover, NH, United States; 2 CairnSurgical, Inc., Lebanon, NH, United States; 3 Department of Radiology, Dartmouth-Hitchcock Medical Center, Lebanon, NH, United States; 4 Department of Biomedical Data Science, Geisel School of Medicine at Dartmouth, Hanover, NH, United States; 5 Department of Surgery, Dartmouth-Hitchcock Medical Center, Lebanon, NH, United States; 6 Department of Radiology and Medical Imaging, University of Virginia Health, Charlottesville, VA, United States

**Keywords:** tumor contrast quantification, BCS planning, breast neoplasm, contrast-enhanced MRI, multimodal MRI, supine breast MRI

## Abstract

**Purpose:**

For surgical guidance applications, supine breast MRI tumor contrast should be non-inferior to prone MRI, currently considered the standard-of-care. However, comparing image contrast quantitatively between different MRI sequences and breast orientations presents a significant challenge. Herein, we present a method for quantitatively comparing regional tumor contrast in the prone and supine breast MRI orientations for the purpose of tumor localization, and we apply this framework to assess the performance of two investigational supine scans (i.e. independent and prone-to-supine, P2S) compared to diagnostic prone MRI.

**Methods:**

Patient tumors from two studies (NCT03573804, NCT03573661) were outlined slice-by-slice by a breast radiologist using Gd-enhanced, T1-weighted MRI. Image data were derived from subjects undergoing standard-of-care prone imaging (n = 78), independent supine imaging (n = 17), and P2S supine imaging (n = 61). Normalized tumor contrast was computed between the segmented tumor and neighboring normal tissue regions and compared for statistical differences amongst cohorts and non-inferiority to prone MRI.

**Results:**

The independent supine cohort possessed non-inferior tumor-to-fibroglandular contrast compared to prone (p = 0.002), while tumor-to-fibroglandular contrast from the P2S supine cohort was found inferior to the prone cohort. However, both investigational supine scans produced non-inferior tumor-to-adipose contrast when compared to prone MRI (p< 0.001 in both cases).

**Conclusions:**

Regional contrast between tumor and surrounding fibroglandular tissue suffered at later timepoints observed in the P2S supine study, resulting in inferior tumor contrast. However, when contrast-enhanced supine breast MRI is acquired independently, ratiometric comparisons indicate that tumor contrast is non-inferior to prone MRI.

## 1 Introduction

The most sensitive imaging examination for breast cancer diagnosis remains bilateral contrast-enhanced prone breast MRI (1–4). Standard-of-care (SoC) contrast-enhanced prone MRI allows three-dimensional visualization of breast tissue in a pendent position and detects invasive cancer and enhancing ductal carcinoma *in situ* (DCIS) with excellent sensitivity (5–7). Despite these advantages, prone MR images have not been shown to improve outcomes for breast-conserving surgery (BCS), as demonstrated in both the COMICE and MONET clinical trials (8, 9). This finding may be due to the significant deformation of the breast tissue that occurs between the imaged prone and surgical supine positions, which changes orientation and position of the tumor significantly (10–12).

As one approach to address this deformation challenge, efforts to model breast tissue deformation from prone-to-supine positions have been underway (13–15). However, these methods are often computationally expensive and often do not produce consistent, accurate results across the broad array of breast sizes and tissue densities encountered in real world settings. As an alternative solution, supine breast MRI offers tumor localizing information in an anatomically-relevant surgical position (16–20). This approach has been leveraged in the development and clinical testing of several image-guidance approaches, with demonstrated potential for decreasing positive margins in BCS (21–23).

Selective tumor contrast uptake and kinetics also play a critical role in accurate tumor localization and segmentation of its 3D extents, as required for MR image-guided surgery applications (21–30). Thus, in order to reproduce the contrast kinetics of the gold standard diagnostic prone exam, supine breast MR images are generally acquired independently in a separate imaging session (17–20), thus requiring two MRI appointments on different days. In an effort to reduce expense, time, and scheduling constraints inherent in two separate (prone and supine) imaging sessions, we developed and clinically tested a protocol to acquire supplemental supine MRIs directly after SoC diagnostic prone MRIs (i.e. prone-to-supine, P2S, imaging) using a single contrast injection during the same imaging appointment (The Prone to Supine Breast MRI Trial, #NCT03573804).

While performing a supplemental supine breast MRI immediately after a diagnostic prone breast MRI in one imaging session provides clear logistical benefits, the resulting image contrast observed in supplemental supine scans using the P2S technique has yet to be quantified or compared to SoC prone MRI or independent supine MRI (10, 12). These comparisons are not straightforward as absolute contrast-enhanced MRI images are not globally comparable, due to variations in MR imaging equipment, patient-specific scan settings, choice of imaging coils used etc. As such, metrics for quantitative comparisons between diagnostic prone and supine MR image quality have not been adequately explored in prior studies (19).

To address this issue, we developed and applied a method for quantifying ratiometric image contrast associated with tumor localization (relative to adjacent background parenchymal enhancement) across multiple breast MRI exams using data from two investigational supine imaging protocols and a SoC prone imaging study. We applied the approach to evaluate the inferiority/non-inferiority of supine MRI tumor contrast under these two clinical exam settings relative to SoC prone MRI: 1) independent supine MRI obtained with contrast injection during an independent imaging session on a separate day and 2) P2S supine MRI obtained directly after the prone MRI with delayed contrast from the prone exam injection.

## 2 Methods

All image data utilized in this study were obtained from consenting subjects enrolled in one of two prospective experimental studies (#NCT03573804; #NCT03573661) investigating technologies for supine MRI-guided breast-conserving surgery. The experimental protocols for both studies received approval from Dartmouth’s Committee for the Protection of Human Subjects. Prior to participation, informed consent was obtained from all subjects. Additionally, de-identified MRI image data were shared with CairnSurgical, Inc. under a data sharing agreement.

### 2.1 Breast MRI technique

Breast MRI data were acquired on either a 1.5T Siemens scanner (MAGNETOM Aera or MAGNETOM Symphony; Siemens Medical Solutions, Malvern, PA, USA), a 3.0T Siemens scanner (MAGNETOM Prisma or MAGNETOM Skyra; Siemens Medical Solutions, Malvern, PA, USA), or a 1.5T GE MRI scanner (SIGNA™ Explorer; General Electric Company, Philadelphia, PA, USA).

For prone MRI, SoC prone MRI imaging techniques and sequences were performed using either an ultrafast 3D gradient echo (GE) sequence (VIBRANT FS) on 1.5T GE scanners, or a spoiled GE sequence (3D FLASH) with spectral (Quick-FatSat) fat suppression on 1.5T and 3.0T Siemens scanners.

For supine MRI, as previously described elsewhere (28), patients are positioned supine on the scanner with their ipsilateral arm kept parallel to their body. Foam pads are placed on the patient’s chest to support the flex body coil and minimize breast compression so that it remains in its native position as expected in surgery.

For supine image acquisition, axial T1 fat-saturated scans were acquired using either a fast-spoiled gradient echo sequence (3D LAVA-Flex) on GE 1.5T scanners, or a spoiled gradient echo sequence (3D FLASH) in combination with either spectral (Quick-FatSat) or inversion-recovery (SPAIR) fat suppression techniques on Siemens 1.5T and 3.0T scanners. Breath holds were not utilized because early feasibility studies found such efforts to be counterproductive and not reproducible across all machines and patients. Instead, the chosen supine MRI sequences relied on rapid acquisition and signal averaging techniques. Additional scanning specifications for the supine MRI sequences utilized as part of this study are provided in **Table 1** for both GE and Siemens scanners.

**Table 1:** Supine breast MRI scan parameters.

System	Sequence name	TR	TE	FOV	Matrix	Phase direction	Flip angle	NEX	Slice thickness	Coils
GE 1.5T	3D LAVA-Flex	6.5 ms	3.1 ms	220–280 mm	200x200	A-P	10	3	2 mm	GEM Flex Coil 16-L Array
Siemens 1.5T	3D FLASH	6.4 ms	2.6 ms	220–280 mm	384x90	R-L	10	1	2 mm	18-channel body array + spine coil (Body 18)
Siemens 3.0T	3D FLASH	4.2 ms	2.0 ms	220–280 mm	320x80	R-L	10	2	2 mm	18-channel body spine array coil (Body 18)

For contrast imaging, intravenous Gd-agent Dotarem^®^ (gadoterate meglumine) was administered at the prescribed dose of 0.2 mL/kg. In the prone and independent supine MRI protocols, pre-Gd images were obtained prior to contrast administration, and post-Gd images were obtained 45s post Gd-administration. In the P2S protocol, post-Gd supine images were acquired on average 23.0 ± 2.7 min. after prone MRI contrast administration.

The P2S cohort scans were all performed in one location, using the same scanner and scanning protocol, specified in **Table 1**. The independent supine cohort was acquired across multiple sites using GE 1.5T, Siemens 1.5T, or Siemens 3.0T systems; however, all scans employed either 3D LAVA-Flex (GE) or 3D FLASH (Siemens) sequences, which are closely matched in terms of contrast weighting and resolution. While minor variability in vendor-specific implementation is unavoidable, the core imaging parameters were similar. Image analysis was performed on the first acquired post-Gd T1-weighted, fat-saturated scans; however, the prone and supine MRI sequence parameters differed slightly, demonstrating the need for exam-independent image comparison methods.

### 2.2 Study cohorts

#### 2.2.1 Prone cohort

This MRI dataset (n = 78) resulted from patients enrolled in two studies: 61 subjects participating in The Prone to Supine Breast MRI trial (#NCT03573804) and 17 individuals entered in a Pilot Multi-Institutional study (#NCT03573661). All patients demonstrated at least 1 cm of disease as measured on mammography and/or ultrasound with a histologic diagnosis of either invasive breast cancer or ductal carcinoma *in situ* (DCIS). The prone cohort received SoC prone MRI, either immediately prior to P2S supine imaging (n = 61) or on a separate day prior to the supine MRI exam (n = 17).

#### 2.2.2 Independent supine cohort

This MRI dataset (n = 17) resulted from patients enrolled in a Pilot Multi-Institutional study (#NCT03573661) who had >1 cm mass and received SoC prone and supine MRI on separate days/imaging sessions.

#### 2.2.3 P2S supine cohort

This MRI dataset (n=61) resulted from patients enrolled in The Prone to Supine Breast MRI trial (#NCT03573804) who had >1cm mass and received SoC prone MRI immediately prior to a supine MRI exam. The P2S imaging protocol involved acquisition of SoC contrast-enhanced prone MRI, followed by turning the patient over to the supine position to acquire additional supine images (P2S supine) during the same imaging session and without additional contrast injection. Average time from contrast administration (prone) to start and end of the dynamic supine sequence was 23.0 ± 2.7 min. and 29.3 ± 2.9 min., respectively. SoC prone MRI data from these subjects are included in the prone cohort.

### 2.3 Segmentation methods

**Figure 1:**
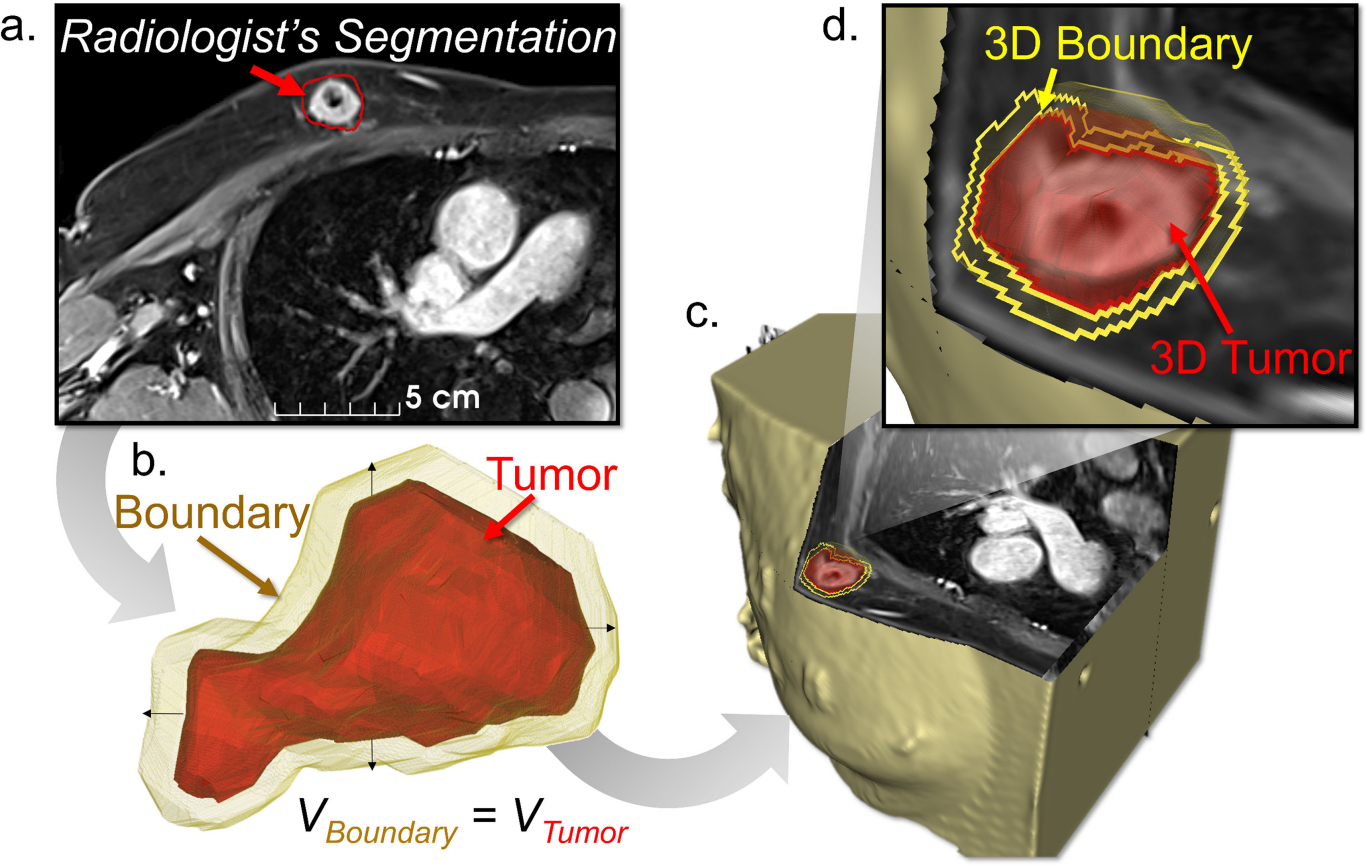
**(a)** Radiologist’s slice-by-slice segmentation of tumor region, **(b)** 3D rendered tumor model and surrounding boundary regions, **(c)** Segmentations overlaid onto MRI volume with **(d)** tumor and boundary voxel regions identified.

A breast radiologist with 21 years of experience outlined tumor edges on contiguous axial MRI slices acquired with subjects positioned in prone and supine positions as illustrated in **Figure 1a**. Both mass and non-mass enhancing (NME) regions were included in the radiologist’s segmentation. In cases of multiple disparate lesions, only the primary lesion was segmented. A 3D tumor model was constructed using 3D Slicer (Version 4.11, www.slicer.org) on the radiologist-segmented MRI obtained from Ambra (Ambra Health Inc., New York, NY). Additional image analysis was performed in MATLAB (v.2021a).

To define tumor boundary regions, adipose and fibroglandular tissue directly surrounding the segmented tumor were selected with a margin thickness that ensured equivalent volumetric comparisons between tumor and boundary regions (**Figure 1b**). Utilizing the MRI signal units within the 3D tumor and boundary regions (as illustrated in **Figures 1c, d**), we computed the normalized tumor-to-boundary contrast as follows:

(1) $$Normalized\;Contrast=\frac{I_{Tumor}-I_{Boundary}}{I_{Tumor}+\;I_{Boundary}}$$

where $I_{Tumor}$ and $I_{Boundary}$ represent the average intensities within the selected voxel regions of the tumor and boundary, respectively. Dividing MR intensity differences by their sum $\left(I_{Tumor}+I_{Boundary}\right)$ ensured contrast values ranged from [-1, 1] and bounded the measurements within a constant window for global comparisons. In effect, we compute Michelson contrast, a commonly used metric for quantifying normalized contrast between two region-based signal intensities (31, 32). Clinically, this metric provides a standardized, normalized measure of how clearly anatomical structures or lesions can be distinguished from surrounding tissues, with higher values indicating better delineation. In this study, we applied the Michelson contrast formula (Equation 1) to calculate normalized tumor−to−boundary contrast as an overall measure of tumor visibility relative to adjacent boundary tissue, accounting for contributions from both adipose and fibroglandular components within that region. We then stratified the analysis by tissue type to separately report tumor-to-adipose and tumor-to-fibroglandular normalized contrast values.

**Figure 2:**
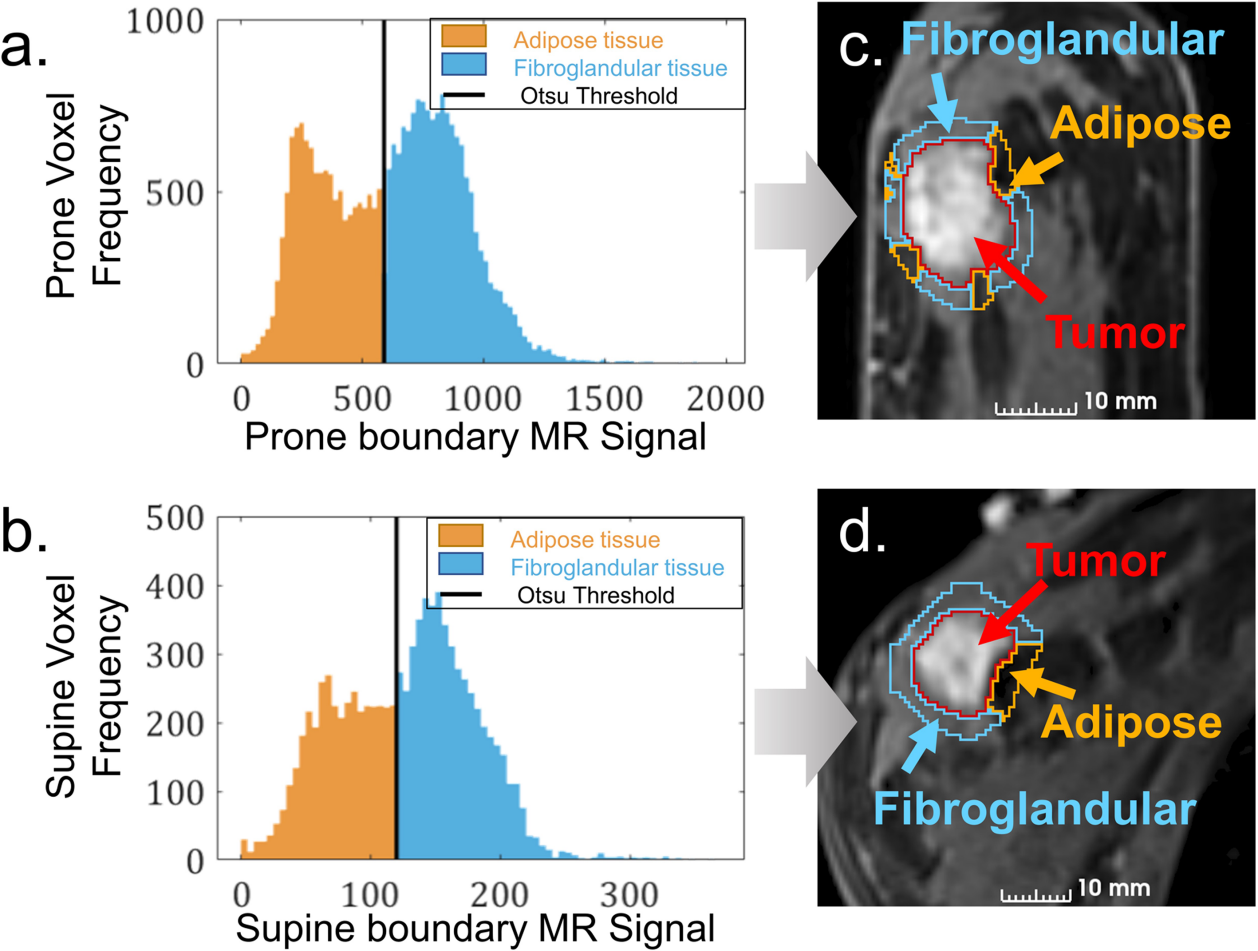
Exemplary boundary tissue MRI signal intensity histograms with Otsu thresholding to distinguish adipose from fibroglandular in **(a)** prone and **(c)** supine orientations. Resulting segmentations distinguish tumor-surrounding adipose (orange) from fibroglandular tissues (blue) in the **(c)** prone and **(d)** supine MRI.

Boundary regions were classified as adipose or fibroglandular using Otsu’s method for intensity thresholding (33) (See **Figures 2a, b**). In some cases, fibroglandular segmentations included pectoral muscle located within the boundary region. Resulting segmentations appear in **Figures 2c, d**. All segmentations were confirmed by the lead breast radiologist’s visual inspection. Using segmented fibroglandular and adipose regions, normalized tumor-to-fibroglandular contrast was calculated according to Equation 1 (mean tumor intensity minus mean fibroglandular intensity divided by their sum), and similarly, tumor-to-adipose contrast.

Normalized tumor-to-fibroglandular and tumor-to-adipose contrast values were compared between the prone, independent supine, and P2S supine MRI datasets. Contrast comparability was based on these ratiometric contrast measures in part to mitigate the impact of differences in imaging protocols and scanners across the study cohorts. To estimate the amount of fibroglandular tissue surrounding each segmented tumor volume, a compositional percentage was calculated as volume of fibroglandular tissue in the boundary region divided by the total boundary volume. Signal−to−noise ratios (SNR) and contrast−to−noise ratios (CNR) were also computed and stratified by tumor−to−fibroglandular and tumor−to−adipose tissue comparisons. In addition, Receiver Operating Characteristic (ROC) area−under−the−curve (AUC) analyses were performed to provide additional quantitative measures of imaging performance when comparing the prone, independent supine, and P2S supine imaging cohorts. All attempted segmentations were completed by a single radiologist with considerable experience in reading supine MRI and ‘segmenting’ tumors for surgical guidance. Because the P2S technique is new and supine breast MRI is not widely adopted clinically, relying on the most experienced radiologist helped minimize operator-related error, which is appropriate at this early stage of investigation.

### 2.4 Statistical analysis

A series of two-sample t-tests was performed amongst the three MRI datasets (prone, independent supine, and P2S supine) for tumor-to-fibroglandular and tumor-to-adipose contrast measurements with α = 0.05 and assumed unequal variances. To address sample size limitations, a *post-hoc* power (PHP) analysis was applied to generate 95% confidence intervals (CIs) and Power for each statistical test. All sample populations were confirmed to be normally distributed using the One-sample Kolmogorov-Smirnov test. Sample sizes for each cohort were determined by the design and enrollment of the two prospective clinical studies (#NCT03573804 and #NCT03573661) from which data were derived. Although the independent supine cohort was smaller, sample size disparities were addressed by using nonparametric Mann–Whitney U tests for all pairwise comparisons. These tests do not assume normality, equal sample sizes, or equal variances and are well suited for the distribution characteristics observed in our data.

Differences between the two investigational supine scans (independent supine and P2S supine) when compared to prone were calculated with 95% CI. Non-inferiority tests were conducted on independent supine and P2S supine in which the non-inferiority margin was set to a difference of -0.1, representing a 10% decrease in normalized contrast. Using a two-sample, one-tailed t-test (α = 0.025), the null hypothesis of inferiority was rejected if p< 0.025 and the 95% CI extended below the non-inferiority margin.

Tumor-to-boundary contrast was not statistically compared across cohorts because it is not a reliable measure due to varying compositions of adipose and fibroglandular tissue in the boundary regions across patients. To examine the dependence of tumor-to-boundary contrast on boundary tissue composition, we looked at the correlation between tumor-to-boundary contrast and fibroglandular boundary composition (%) in paired prone and P2S supine MRI datasets (n = 61) through Pearson’s correlation coefficient (r) and its 95% CI. Additional analyses were performed to examine associations between P2S tumor contrast and tumor size metrics (volume and maximum extent), tumor shape characteristics (sphericity and isocentricity (34)), and disease subtype to determine whether specific morphologic or histopathologic characteristics were predictive of reduced contrast.

## 3 Results

**Figure 3:**
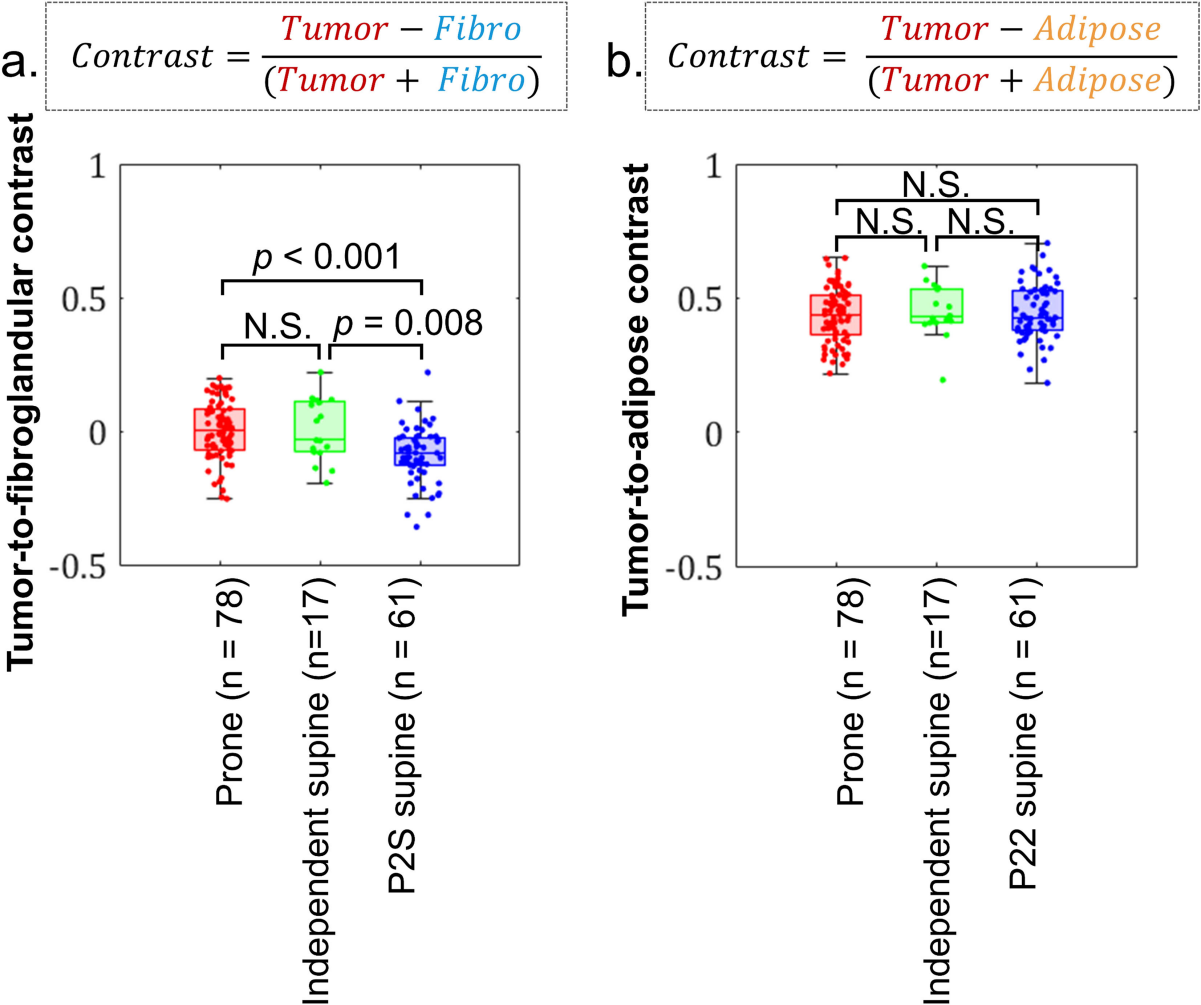
Boxplot distributions and two-sample, t-test statistical outcomes for comparisons between prone, independent supine, and P2S supine image data for **(a)** tumor-to-fibroglandular and **(b)** tumor-to-adipose contrasts.

Distributions of tumor-to-fibroglandular and tumor-to-adipose contrast for prone, independent supine, and P2S supine datasets are shown in **Figure 3**. P2S supine image data exhibited lower tumor-to-fibroglandular contrast compared to others (**Figure 3a**). Using a two-sample, two-tailed t-test, mean P2S supine tumor-to-fibroglandular contrast was different statistically from the prone (μ_diff_ = -0.09, 95% CI = [-0.12, -0.05], p< 0.001, PHP>0.99) and independent supine cohort (μ_diff_ = -0.09, 95% CI = [-0.16, -0.03], p=0.008, PHP = 0.80). No statistical differences were found amongst tumor-to-adipose contrast comparisons amongst the three datasets (**Figure 3b**).

**Figure 4:**
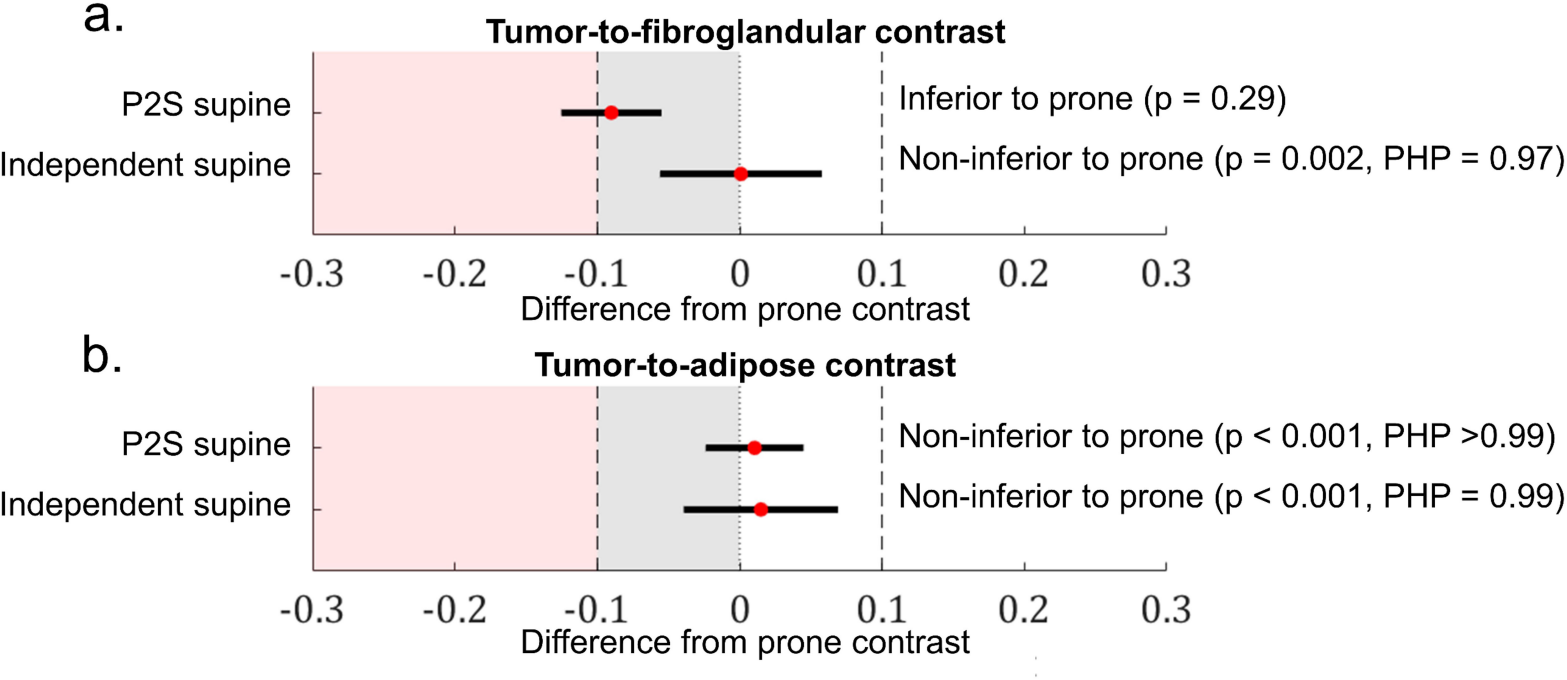
Forest plots exhibiting differences from the prone contrast values for P2S supine and independent supine measurements of **(a)** tumor-to-fibroglandular and **(b)** tumor-to-adipose normalized contrasts. The non-inferiority margin (grey) was set to be a difference of 0.1 from the prone contrast measurement.

Using one-sided, two-sample t-tests to test for non-inferiority (α = 0.025) and associated power calculations for 95% CI, the p-value and *post-hoc* power (PHP) calculations for non-inferiority of independent supine and P2S supine contrast values relative to prone measurements are displayed in **Figure 4** as forest plots with the 95% CIs for sample differences represented as solid black lines in the figure.

The non-inferiority margin [-0.10, 0] is depicted as the gray region in the plots of **Figure 4**. Across both contrast metrics, the independent supine contrast was found to be statistically non-inferior to prone contrast within a margin of 0.1 normalized contrast units. For tumor-to-fibroglandular contrast (**Figure 4a**), the P2S supine contrast was found to be inferior to prone, crossing over the non-inferiority margin. The P2S supine contrast values fell above the non-inferiority margin for tumor-to-adipose contrast (p< 0.001, PHP > 0.99); thus, we reject the null hypothesis of inferiority in this case.

**Figure 5:**
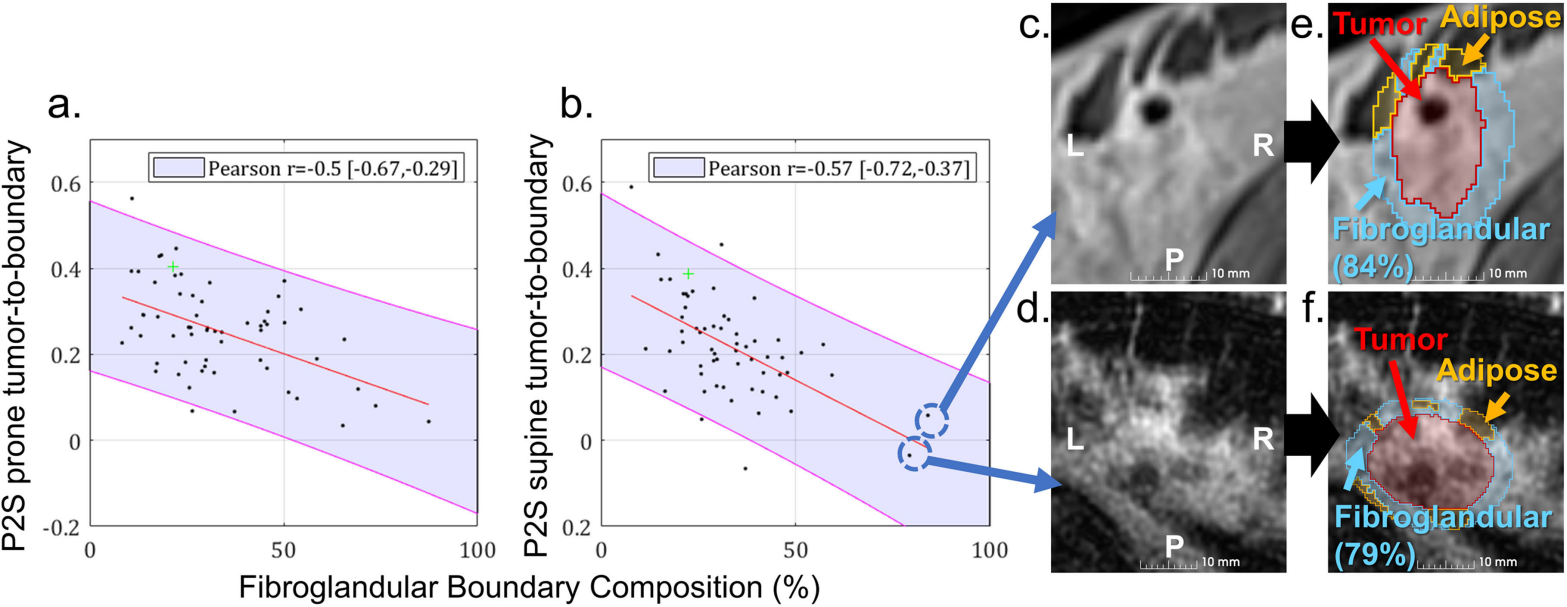
Correlation between tumor-to-boundary contrast and fibroglandular boundary composition (%) for the P2S cohort’s **(a)** prone MRI and **(b)** P2S supine MRI datasets (n = 61). **(c, d)** T1-weighted MRI examples from the P2S supine dataset exhibiting high fibroglandular composition (%) around the tumor region with **(e, f)** overlaid labels.

As another interesting finding, negative correlations were observed between fibroglandular boundary composition (%) and resulting tumor-to-boundary contrast (**Figure 5**). The linear relationships for the paired prone (Pearson’s r = -0.50, [-0.67, -0.29]) and P2S supine (Pearson’s r = -0.57 [-0.72, -0.37]) datasets exhibited similar negative trends. Cases with high amounts of fibroglandular tissue in the tumor boundary often resulted in reduced tumor-to-boundary contrast as demonstrated in **Figures 5c, d** with 84% and 79% fibroglandular boundary composition, respectively. Overall, the strongest association with tumor contrast was observed for fibroglandular boundary composition. A modest positive correlation was identified between maximum tumor extent and tumor-to-boundary contrast in the P2S supine cohort (Pearson’s r = 0.32, [0.07, 0.53]). Similarly, a slight positive correlation was observed between tumor volume and tumor-to-boundary contrast in both the paired prone and P2S supine datasets (Pearson’s r = 0.29, [0.04, 0.51] and r = 0.28, [0.03, 0.50], respectively). No statistically significant associations were found between tumor contrast ratios and tumor shape characteristics (isocentricity or sphericity) or disease subtype.

**Figure 6:**
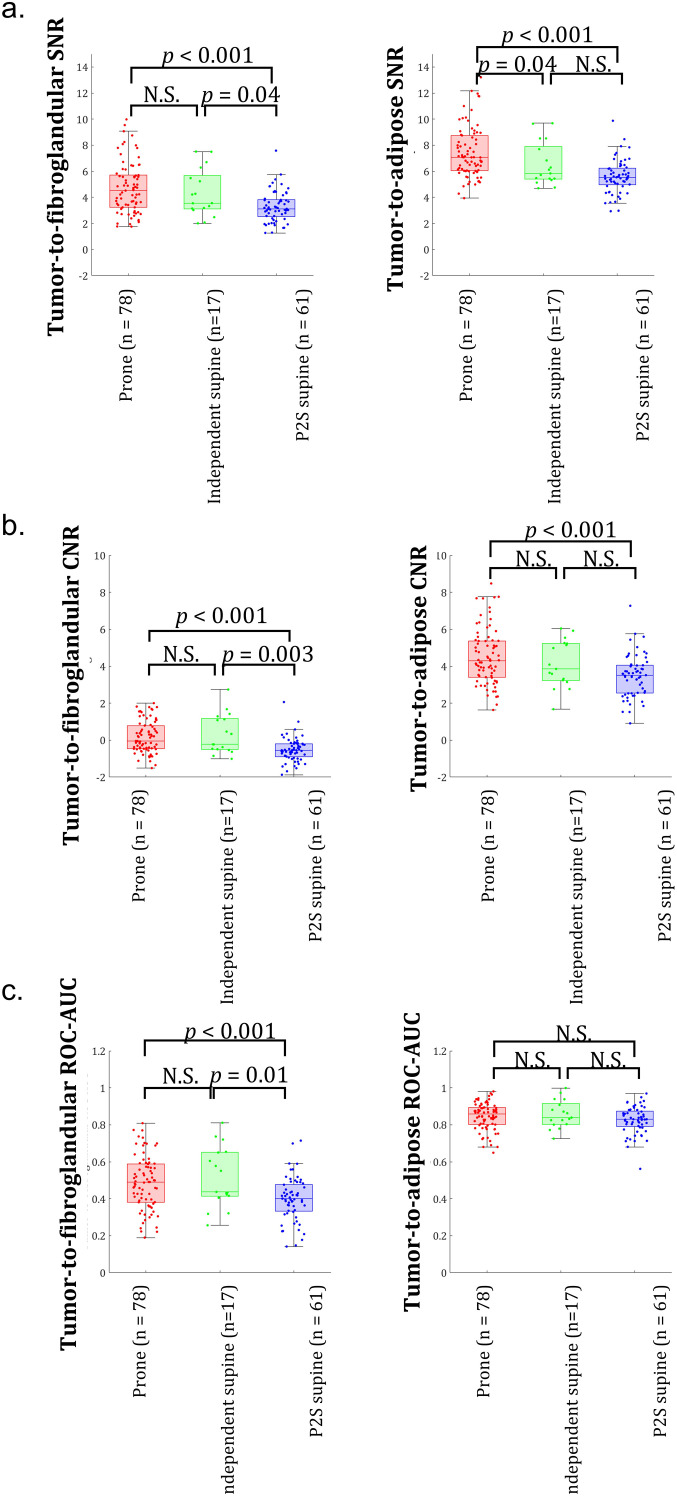
Boxplots of image-derived performance measures for tumor-to-fibroglandular contrast (left) and tumor-to-adipose contrast (right) for prone, independent supine, and P2S supine cohorts: **(a)** Signal-to-Noise Ratio, SNR, **(b)** Contrast-to-Noise Ratio, CNR, and **(c)** Receiver Operator Characteristic (ROC) area under the curve (AUC). Statistical differences in means were evaluated using pairwise Mann-Whitney-U tests (nonparametric). P values are shown for comparisons that reached statistical significance. NS indicates non-significant results (p > 0.05).

**Figure 6** presents boxplots of SNR (6a) and CNR (6b) values for tumor-to-fibroglandular and tumor-to-adipose contrasts across prone, independent supine, and P2S breast MRI image data. ROC AUC analyses for these tissue contrasts are also presented in **Figure 6c**. Statistical differences in the image-derived quantities shown in **Figure 6** (prone, independent supine, and P2S data) were assessed using pairwise Mann–Whitney U tests. P values are reported for significant differences, with “NS” indicating non-significant results (P > 0.05).

## 4 Discussion

While previous studies have reported on supine MRI feasibility (17–20), prone vs. supine MRI characteristics (11, 18, 19), and prone-to-supine (P2S) MRI study results (10, 12), tumor contrast has yet to be systematically compared between prone and supine breast MRI scans. To address this gap, we utilized ratiometric contrast measurements to compare prone and supine breast MRI acquisitions and assess acceptability of investigational supine MRI for the first time. Additional image-derived performance measures, namely SNR, CNR, and ROC AUC, were also evaluated for both tumor-to-fibroglandular and tumor-to-adipose contrasts across prone, independent supine and P2S supine MRI datasets. Our results showed no statistical difference in tumor contrast between prone and supine breast MRI when acquired separately (independent supine MRI) and suggest that breast orientation change from prone (for SoC imaging) to supine (for surgical planning) is likely to be acceptable, clinically, for determining MRI-defined tumor extent in the supine position. These findings are supported by SNR, CNR, and AUC analyses, which also showed no statistical difference in tumor-to-fibroglandular contrast between independent supine and prone breast MRI.

While supine MRI has been demonstrated to produce images of excellent quality using clinically available MRI machines, coils, and sequences (17, 19, 25, 34); supine MRI is not likely to be a replacement for diagnostic prone MRI. From a diagnostic perspective, the prone pendant breast position is advantageous for examining disease extension with little to no respiratory motion artifacts. On the contrary, respiratory motion during ungated supine MRI scans can cause streaking artifacts in the phase encode direction, directly resulting in displaced tissue signal and degraded image quality (17).

Although motivation to perform prone-to-supine MRI within a single imaging session is strong, the sufficiency of tumor contrast in supplemental supine scans for tumor margin delineation has not been quantified previously. Amongst the two published P2S studies (10, 12), both reported on observed lesion displacement from prone-to-supine patient positioning, but did not otherwise examine tumor contrast. Aribal et al. (12) did investigate the radiologists’ ability to detect lesions in supine MRI (12). However, detecting tumor vs. determining its extent are different tasks, the latter requiring high fidelity in tumor margin delineation, which can be evaluated quantitatively by assessing tumor contrast. Our approach was designed to assess image contrast as it relates to segmenting tumor extent in a 3D volume – an important step in surgical planning for optimal breast conserving surgery with negative margins (22).

P2S supine MRI (without additional contrast injection after the prone MRI) demonstrated inferior tumor contrast relative to SoC prone tumor contrast, with lower tumor-to-fibroglandular contrast found in the P2S supine MRI data. Additional image-derived performance assessments, including SNR, CNR and ROC AUC, are consistent with this result. These metrics showed statistically significant differences between independent supine and P2S image data for tumor−to−fibroglandular contrast, but not for tumor−to−adipose contrast. This suggests that the limitations of P2S imaging arise from reduced differentiation of tumor from adjacent fibroglandular tissue compared to independent supine breast MRI. Loss of contrast at later time points (average delay time = 23 min.) may be explained by known Gd-kinetics in which fibroglandular tissue often shows persistent Gd-uptake while tumorous regions washout the Gd-based contrast in the later phases of image acquisition (35, 36). Based on these results, P2S is unlikely to be suitable for surgical planning—at least with the imaging sequences used here—as tumor-to-fibroglandular contrast does not appear sufficiently robust across the range of breast parenchymal patterns encountered in clinical practice to reliably ensure tumor visibility.

Unlike the P2S supine image data, independent supine MRI (acquired in a separate imaging session) demonstrated non-inferior tumor-to-fibroglandular contrast compared to prone MRI, indicating that the independent supine MRI protocol is a viable option for preoperative surgical planning. Recent clinical data (23) further support this, showing that independent supine MRI in conjunction with supine MRI-based surgical guidance significantly reduced positive margin rates in breast-conserving surgery (23). These findings suggest that normalized tumor-to-fibroglandular contrast may serve as a valuable reference metric for evaluating new and/or improved supine MRI protocols or image analysis methods. The values reported here may also establish a baseline for the supine MRI contrast performance required to impact surgical outcomes. Moving forward, the ratiometric contrast approach used here is a powerful tool for examining and comparing image contrast amongst multiple MRI sequences and breast exam protocols independent of patient orientation or image sequence.

While supine MRI achieves tumor contrast comparable to prone MRI when acquired separately, patient and clinical care demands associated with multiple visits suggest a single imaging session solution is needed. Given these preliminary prone-to-supine results, a secondary prone-to-supine study is underway which utilizes two boluses, one before prone and one before supine acquisitions, in a single imaging exam session (ClinicalTrials.gov, # NCT00159939). Other approaches should also be explored to meet the clinical need for efficient, high-fidelity, and integrated supine MRI within the SoC imaging workflow. For example, recent advancements in MRI-based radiomics highlight the potential of radiomics-informed and AI-assisted methods for breast tumor segmentation and characterization, which could be adapted for supine imaging. Zhang et al. (37) developed an nnUNet-based radiomics pipeline for multi-sequence breast MRI, achieving high segmentation accuracy (Dice score up to 0.86) and excellent differentiation between benign and malignant lesions through feature extraction from tumor regions (37). Other groups (Yu et al., 2024) have explored the integration of intra- and peritumoral radiomics with deep learning, notably improving classification performance compared to expert radiologists when applied to DCE-MRI (38). Liao et al. (39) utilized DCE-MRI radiomics nomograms, combining imaging and clinical features, to successfully predict pathological upgrades in high-risk lesions, with AUCs approaching 0.91 in training and 0.87 in validation cohorts (39). In the neoadjuvant chemotherapy setting, Pesapane et al. (40) showed that peritumoral radiomics features derived from DCE-MRI correlated with pathological complete response, outperforming traditional clinical markers (AUC ~0.76) (40). Similarly, Han et al. (2024) applied machine learning to MRI radiomics data to predict lymphovascular invasion in HER2-positive breast cancer, achieving AUCs of 0.96 and 0.95, thereby exceeding conventional radiologist-based assessments (41). Collectively, these studies underscore the growing role of MRI radiomics and AI-based tools in enhancing breast tumor detection, segmentation, and risk stratification. Integrating such approaches into clinical workflows has the potential to improve diagnostic and prognostic precision and could be adapted to supine MRI—or even P2S—for future surgical planning applications.

These findings support a pragmatic approach for incorporating supine MRI into breast-conserving surgery (BCS) planning when MRI-defined tumor extent is clinically important (e.g., nonpalpable disease (42), extensive ductal carcinoma *in situ* components (43), lobular histology (44, 45), or discordant ultrasound/mammography findings (45)). When independently acquired supine MRI provides tumor-to-fibroglandular contrast comparable to the diagnostic prone exam, surgeons can plan resections using a dataset that is already in an anatomically relevant operative position. In practice, these images may improve the reliability of translating three-dimensional tumor extent to surgical decisions such as incision placement, resection orientation, anticipated margin targets, and oncoplastic strategy, and can support patient-specific guidance (e.g., skin-surface targeting, bracketing of the MRI-defined volume, and specimen orientation) without relying on uncertain prone-to-supine deformation assumptions.

From a workflow perspective, supine MRI is best positioned as an adjunct to existing breast imaging exams rather than a replacement for diagnostic prone MRI. A feasible supine MRI integration model is: (1) standard diagnostic prone MRI for staging and extent, followed by (2) a dedicated, short supine contrast-enhanced acquisition either alone or possibly in the same session (if timed to optimize enhancement or with a second contrast injection), and (3) multidisciplinary review in which the supine dataset is used for operative targeting in conjunction with standard localization methods (wire, seed, radar/magnetic, or skin marking) and/or patient-specific surgical aids. The present data also suggest an actionable workflow decision point: if a same-day prone-to-supine (P2S) protocol cannot maintain tumor-to-fibroglandular contrast near the early post-contrast phase, it is unlikely to provide sufficiently robust tumor boundary definition for consistent surgical planning across breast densities, and an independent supine acquisition (or an alternative single-session protocol that re-establishes early enhancement) should be preferred.

Alternative strategies aim to transform diagnostic prone MRI into an estimated surgical supine configuration using either surface-driven/intensity-based deformable registration or physics-based biomechanical modeling (often finite element methods under gravity and boundary constraints). For example, surface-based workflows have combined Laplacian deformation, iterative point registration, and B-spline models to estimate tumor location in the surgical pose using prone MRI plus a supine surface measurement (46). Biomechanical methods can incorporate tissue heterogeneity and patient-specific boundary conditions to predict large deformations and co-locate tumor information across imaging positions and modalities (47). These methods may reduce the need for a separate supine contrast-enhanced visit or even a combined prone-to-supine single session exam, but they introduce additional sources of uncertainty (model assumptions, boundary conditions, tissue property estimation, and the need for robust validation across breast sizes and densities) that can complicate clinical deployment (48).

More recently, data-driven deformable registration approaches, including deep learning models, have been adapted to prone-supine breast MRI registration and can offer rapid inference once trained, potentially enabling near-real-time pose correction in planning pipelines (49). In parallel, improved automated segmentation of breast and surrounding tissues across prone and supine acquisitions may strengthen both biomechanical and learning-based deformation methods by providing more reliable tissue labels and constraints (50). In the near term, independently acquired supine MRI provides a direct, anatomically relevant reference for surgical planning and can also serve as a valuable ground truth for training for either a single session combined prone-to-supine breast MRI exam that maintains contrast in the supine portion of the acquisition or validating registration or deformation models intended to eliminate the second imaging session completely.

Some important limitations and caveats associated with the study exist. For example, patient−specific factors such as demographics, disease and lesion type, and receptor status were not examined but may represent important variables to consider in future studies. In addition, cohort sizes for the imaging procedures analyzed were different (independent supine n = 17, P2S supine n = 61, and prone n = 78) due to differing sizes of the two clinical trials from which image data were derived. While sample size disparities were addressed by using nonparametric Mann–Whitney U-tests for all pairwise comparisons, generalizability of these findings remains limited, and follow up studies with larger cohorts are needed to draw more definitive conclusions. Although all segmentations were performed by a single radiologist, albeit the most experienced in supine MRI and therefore minimizing operator−related error in this study, future work will need to address intra-observer variability for the technique to progress toward broader clinical adoption. Finally, although preliminary data suggest that supine MRI can improve surgical outcomes (23) and tumor-to-fibroglandular contrast from independent supine breast MRI may provide a baseline target for future investigational techniques, definitive evidence is lacking that surgical planning or guidance based on prone-to-supine (P2S) imaging or related methods will improve surgical outcomes. Further investigation is needed to establish this link.

## 5 Conclusion

The ratiometric contrast method presented herein enabled quantitative comparisons of regional tumor contrast amongst standard-of-care prone and investigational supine breast MRI datasets. When acquired separately, supine MRI produced non-inferior tumor contrast compared to prone MRI. Confirming non-inferiority of supine breast MRI contrast for the first time increases confidence that the breast orientation change from prone (for SoC imaging) to supine (for surgical planning) can have a positive clinical impact on BCS outcomes (22, 23, 28). However, regional contrast between tumor and surrounding fibroglandular tissue often suffered at later timepoints in the P2S supine study, resulting in overall inferior P2S supine contrast. This finding suggests that further work is needed to acquire prone and supine images with non-inferior tumor contrast within a single breast MR imaging session.

## Data Availability

The raw data supporting the conclusions of this article will be made available by the authors, without undue reservation.
